# Preoperative oncologic therapy and the prolonged risk of venous thromboembolism in resectable pancreatic cancer

**DOI:** 10.1002/cam4.4397

**Published:** 2022-02-11

**Authors:** Annika Eurola, Harri Mustonen, Nora Mattila, Riitta Lassila, Caj Haglund, Hanna Seppänen

**Affiliations:** ^1^ Department of Surgery Translational Cancer Medicine Research Program Faculty of Medicine University of Helsinki and Helsinki University Hospital Helsinki Finland; ^2^ Department of Coagulation Disorders Faculty of Medicine University of Helsinki and Helsinki University Hospital Helsinki Finland; ^3^ HUSLAB Laboratory Services Clinical Chemistry Helsinki Finland

**Keywords:** long‐term effects, neoadjuvant therapy, pancreatic neoplasms, pancreaticoduodenectomy, preoperative chemoradiation, venous thrombosis

## Abstract

**Background:**

Pancreatic cancer is one of the most prothrombotic cancers. Among patients receiving preoperative chemotherapy followed by surgery, chemotherapy and surgery represent a compound risk for venous thromboembolism (VTE), rendering the postoperative time a period of interest. We aimed to analyze whether preoperative oncologic therapy increases the risk for VTE after surgery and identify which characteristics associate with VTE.

**Methods:**

We first identified patients surgically treated for pancreatic cancer at Helsinki University Hospital between 2000 and 2017, collecting the following data: gender, age at surgery, preoperative medication, body mass index (BMI), preoperative chemo(radio)therapy, tumor size, positive node ratio, perineural and perivascular invasion, tumor grade, surgical technique, postoperative anticoagulation, adjuvant therapy, time of VTE, time of local disease recurrence, time of distant metastasis, and time of death. With a follow‐up period of at least 2 years or until death, we compared a total of 93 preoperative oncologic therapy and 291 upfront surgery patients (*n* = 384, median age 66.5 years).

**Results:**

Preoperative oncologic therapy increased the risk for thrombosis after surgery (hazard ratio [HR] 1.61; 95% confidence interval [CI] 1.03–2.53). The VTE incidence rate remained high for up to 2 years after surgery. BMI ≥30 kg/m^2^, prior anticoagulation, and disease recurrence (*p* < 0.05, respectively) associated with VTE. VTE is also associated with shorter overall survival (HR 3.25; 95% CI 2.36–4.44). In 71.6% (95% CI 60.5–81.1) of patients, VTE was diagnosed after disease recurrence.

**Conclusions:**

Preoperative oncologic therapy represents an independent risk factor for VTE, not only during the immediate postoperative period but up to 2 years after surgery. VTE is associated with obesity, prior anticoagulation, and disease recurrence and diminishes overall survival.

## Graphical abstract

This study compared the risk of postoperative venous thromboembolism in pancreatic cancer patients undergoing either preoperative oncological therapy or upfront surgery. Preoperative oncological therapy represented and independent risk factor for venous thromboembolism, not only during the immediate postoperative period but up to 2 years after surgery.

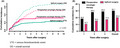

## 1 INTRODUCTION

Venous thromboembolism (VTE) is a well‐established and fatal complication of numerous cancers associated with a shorter survival time and earlier disease recurrence.^1^, ^2^ Cancer carries a fourfold^3^ VTE risk compared with the general population and a five‐ to sevenfold risk compared with noncancer patients.^4^, ^5^ Furthermore, when comparing cancer patients to noncancer patients undergoing similar surgical procedures, cancer patients carry a twofold risk of postoperative deep vein thrombosis and a threefold risk of postoperative fatal pulmonary embolism.^6^

Cancer patients account for more than 20% of all newly diagnosed VTE cases.^7^ Prior studies found that VTE risk is greatest within the first year following a cancer diagnosis.^1^, ^8^, ^9^ Among these patients, VTE is an important cause of morbidity and mortality,^2^, ^10^, ^11^ representing the second leading cause of death.^12^, ^13^

Pancreatic cancer is one of the most prothrombotic cancers,^14^ accompanying the highest rate of VTE with a fourfold VTE risk compared with other cancers and a 15‐fold risk compared with the general population.^3^ Yet, the mechanisms of an increased propensity for coagulation in cancer remain incompletely understood. In pancreatic cancer–related coagulation, the tissue factor is the best‐characterized protein initiating the extrinsic pathway of the coagulation cascade.^15^ Contrary to a healthy endothelium, malignant tissue expresses the tissue factor, which associates with an increased risk of VTE in pancreatic cancer.^16^ A recent study found that an increase in serum tumor marker CA 19‐9 predicts the incidence of VTE in pancreatic cancer patients.^17^ Furthermore, prior studies documented an increased level of Factor VIII in pancreatic cancer.^18^, ^19^

Besides the malignancy, other independent VTE risk factors such as chemotherapy^20^, ^21^; a higher age, particularly over 65 years^22^, ^23^; comorbidities such as renal failure, respiratory disease, cardiovascular disease, acute infection, and obesity^8^, ^23^; immobility^6^; hospitalizations; and cancer‐related surgery^20^, ^24^ further burden cancer patients. Additionally, a prior VTE carries a six‐ to the sevenfold risk of VTE recurrence.^6^, ^25^ Numerous studies corroborate that VTE risk increases with tumor stage, becoming particularly high with distant metastases.^4^, ^6^, ^8^, ^26^, ^27^

Only a few studies have investigated the association between preoperative oncologic therapy and thrombosis in pancreatic cancer. A previous study analyzed 260 resectable or borderline resectable neoadjuvant‐treated pancreatic cancer patients, finding a VTE incidence of 10% during neoadjuvant therapy.^28^ Another study investigated 426 pancreatic cancer patients receiving preoperative chemotherapy followed by surgical resection. Among these patients, 20% experienced a VTE event within 90 days postoperatively.^29^ However, these previous studies included only patients receiving preoperative oncologic therapy, and no comparison with upfront surgery was completed. In addition, they featured rather short follow‐up periods.

This study aimed to investigate the burden of VTE in pancreatic cancer patients receiving preoperative oncologic therapy within 2 years after surgery comparing preoperatively treated and upfront surgery patients. Furthermore, we aimed to identify the factors associated with venous thrombosis and clarify whether VTE associates with a shorter overall or progression‐free survival in preoperatively treated patients.

## 2 PATIENTS AND METHODS

We collected the clinical data for pancreatic ductal adenocarcinoma (PDAC) patients surgically treated at Helsinki University Hospital between 2000 and 2017 (*n* = 493). Among these 493 patients, we excluded 108 based on missing follow‐up data due to moving abroad (*n* = 11), death within 30 days postoperatively (*n* = 5), prior prothrombotic disease (*n* = 2, activated protein C resistance), stage IV or unresectable disease (*n* = 54), or another cancer within 5 years before surgery or during the follow‐up period (*n* = 36). One patient underwent two surgeries and was included based on the first operation only.

The following preoperative data were collected: gender; respiratory and cardiovascular disease; age at surgery; preoperative use of acetylsalicylic acid, statin, or anticoagulation; the reason for the use of anticoagulation; body mass index (BMI); diagnosed tendency for vein thrombosis; and preoperative oncologic therapy. The following postoperative data were collected: tumor size, positive node ratio, perineural and perivascular invasion, tumor grade, surgical technique (vascular reconstruction), postoperative anticoagulation, and adjuvant therapy.

Follow‐up data consisted of the following: occurrence and timing of VTE, local disease recurrence, distant metastasis, and death. Only patients with newly occurring postoperative VTE were included in the VTE analysis. The follow‐up period began at surgery and continued for at least 2 years or until death. Multiple VTEs were noted in 15 patients, only the first of which we included in our analysis. When analyzing the association between disease recurrence and VTE, either local recurrence or distant metastasis was included, whichever occurred first.

The patients who received preoperative chemo(radio)therapy (*n* = 93) had primarily borderline resectable tumors. Of these patients, 26 (28.0%) received radiation. The most frequently used chemotherapies were gemcitabine (*n* = 67, either alone or combined with cisplatin, capecitabine, or paclitaxel) and FOLFIRINOX (*n* = 13). The most frequently used thromboprophylactic regimen was either dalteparin 2500 ky or 5000 ky, or enoxaparin 40 mg. Patients with preoperative conditions such as atrial fibrillation (*n* = 28), heart valve replacement (*n* = 1), or prior thrombotic disease (*n* = 5) treated with anticoagulation received bigger low‐molecular weight thromboprophylaxis doses. Together 11 patients had prior thromboembolism and anticoagulation, but most of them (*n* = 6) had unresectable disease thus they were excluded from the study. The patients treated after 2010 routinely received a 4‐week low‐molecular weight thromboprophylaxis, whereas earlier treated patients received shorter thromboprophylaxis.

### 2.1 Statistical methods

The competing Cox regression analysis and Aalen–Johansen method were used to analyze the associations between preoperative oncologic therapy and VTE. Death was used as a competing risk, and Grey's test was used for statistical significance in the Aalen‐Johansen method. For overall survival analysis, VTE was used as a time‐dependent variable at the time of occurrence, for which we used the Cox regression analysis and the Simon–Makuch methods. The Mantel–Byar test was used for statistical significance in the Simon–Makuch method. The Fisher's exact test, linear‐by‐linear, and the Mann–Whitney *U* test were used to compare nominative and continuous variables, respectively. To analyze the association between thrombosis and disease recurrence, we calculated the difference between recurrence‐free survival time and the proportion of VTE incidence after recurrence. Disease recurrence was determined as distant metastasis or local recurrence. The one‐sample binomial test was used to determine statistical significance in the proportion of VTEs after progression. We used SPSS (version 27, IBM Corp.), R (version 4.0.3, Foundation for Statistical Computing), and STATA/MP (version 16.1, StataCorp LLC) for all statistical analyses.

## 3 RESULTS

### 3.1 Preoperative oncologic therapy and venous thromboembolism

**FIGURE 1:**
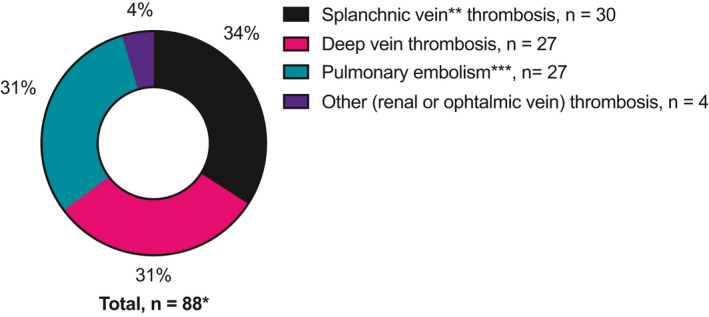
^*^ One patient had both portal vein and deep vein thrombosis, although not simultaneously. ^**^Portal, splenic, or mesenteric vein. ^***^Deep vein thrombosis combined with simultaneous pulmonary embolism was recorded as pulmonary embolism

**FIGURE 2:**
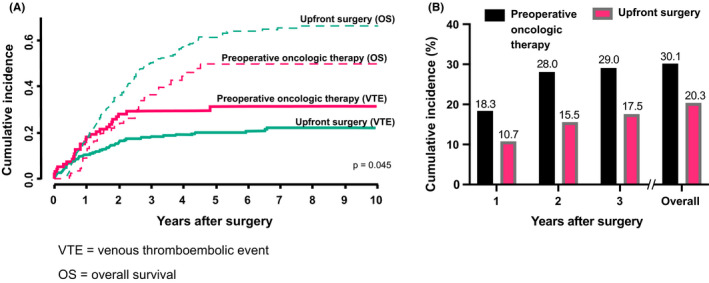
A comparison between preoperative oncologic therapy and upfront surgery patients in cumulative incidence for venous thromboembolism after surgery. (A) Aalen–Johansen analysis describing the statistically higher cumulative incidence rate of venous thromboembolism after surgery in preoperative oncologic therapy patients. Overall survival served as a competing event for venous thromboembolism. (B) A comparison of the cumulative incidence rate of venous thromboembolism between preoperative oncologic therapy and upfront surgery patients 1–3 years after surgery and overall VTE = venous thromboembolic event, OS = overall survival

Altogether, we analyzed 384 (*n* = 93 preoperative chemotherapy and *n* = 291 upfront surgery) patients. Table 1 summarizes the patient characteristics. Among all patients, 87 (23%) experienced VTE during follow‐up. Figure 1 illustrates the distribution of the VTE sites. Overall, the cumulative incidence of VTE after surgery was higher among preoperative chemotherapy patients compared with upfront surgery patients (*n* = 28 [30.1%] vs. *n* = 59 [20.3%]; *p* = 0.049). VTE incidence was highest during the first 2 years postoperatively (Figure 2).

**TABLE 1:** Patient characteristics comparison between the preoperative oncologic therapy and the upfront surgery patients

*n* (%)	Preoperative oncologic therapy	Upfront surgery	*p* value
	93	291	
Gender			
Female	47 (50.5)	145 (49.8)	
Male	46 (49.5)	146 (50.2)	1.000
Age (years) at operation			
≥65	45 (48.4)	177 (60.8)	
<65	48 (51.6)	114 (39.2)	**0.040**
Respiratory disease			
Yes	5 (5.4)	21 (7.2)	
No	88 (94.6)	270 (92.8)	0.641
Cardiovascular disease			
Yes	11 (11.8)	71 (24.4)	
No	82 (88.2)	220 (75.6)	**0.009**
Medication			
ASA			
Yes	19 (20.4)	67 (23.0)	
No	74 (79.6)	224 (77.0)	0.669
Statin			
Yes	19 (20.4)	78 (26.8)	
No	74 (79.6)	213 (73.2)	0.273
Preoperative anticoagulant			
Yes	11 (11.8)	23 (7.9)	
No	82 (88.2)	268 (92.1)	0.290
Postoperative anticoagulant <4 weeks^a^	8 (8.6)	58 (19.9)	**0.007**
Preoperative BMI ≥25 kg/m^2^			
Yes	45 (48.4)	155 (53.3)	
No	48 (51.6)	133 (45.7)	0.404
Missing^b^	3		
Preoperative BMI ≥30 kg/m^2^			
Yes	11 (11.8)	44 (15.1)	
No	82 (88.2)	244 (83.8)	0.498
Missing^b^	3		
Type of surgery			
Pancreaticoduodenectomy	84 (90.3)	245 (84.2)	
Distal pancreatetctomy	6 (6.5)	37 (12.7)	
Total pancreatetctomy	2 (2.2)	9 (3.1)	0.133
Vascular reconstruction			
Yes	55 (59.1)	83 (28.5)	
No	38 (40.9)	208 (71.5)	**<0.001**
Grade			
1	14 (15.1)	54 (18.6)	
2	57 (61.3)	187 (64.3)	
3	20 (21.5)	44 (15.1)	**0.024**
Missing^c^	8		
Perineural invasion			
Yes	65 (70.7)	230 (79.6)	
No	27 (29.3)	59 (20.4)	**0.040**
Missing^c^	3		
Perivascular invasion			
Yes	30 (32.6)	106 (36.7)	
No	62 (67.4)	183 (63.3)	0.454
Missing^c^	3		
Stage			
IA	9 (9.7)	21 (7.2)	
IB	0	45 (15.5)	
IIA	6 (6.5)	6 (2.1)	
IIB	32 (34.4)	126 (43.3)	
III	21 (22.6)	91 (31.3)	**0.030**
Missing^c^	3		
Resection margin			
R0	71 (76.3)	207 (71.9)	
R1	22 (23.7)	81 (28.1)	0.424
Missing^c^	3		
Adjuvant therapy			
Yes	72 (77.4)	212 (72.9)	
No	21 (22.6)	79 (27.1)	0.418
Disease recurrence			
Yes	68 (73.1)	231 (79.4)	
No	25 (26.9)	60 (20.6)	0.251

Bold indicates statistical significance values (*p* < 0.05).

^a^ The routine modality of postoperative thromboprophylaxis before 2010 included anticoagulation while hospitalized.

^b^ Only weight, no height could be found.

^c^ Complete or nearly complete response to preoperative therapy. Few reports lacked data.

Preoperative oncologic therapy emerged as a statistically significant risk factor for VTE after surgery (hazard ratio [HR] 1.61; 95% confidence interval [CI] 1.03–2.53; *p* = 0.037). Figure 2a illustrates the corresponding Aalen–Johansen analysis. In addition, BMI ≥25 kg/m^2^ also represented a statistically significant risk factor for VTE (HR 1.65; 95% CI 1.07–2.55; *p* = 0.024). The other variables examined (age, gender, respiratory or cardiovascular disease, preoperative medication [acetylsalicylic acid or ASA, statin, and anticoagulation], stage, tumor size, grade, perineural invasion, perivascular invasion, vascular reconstruction, type of surgery, resection margin, adjuvant therapy, and anticoagulation <4 weeks) did not increase the risk of VTE (Table S1). We performed a multivariate analysis of factors associated with VTE after surgery. According to the multivariate analysis of factors associated with VTE after surgery, the following factors remained: preoperative oncologic therapy (HR 1.90 [95% CI 1.167–3.082]; *p* = 0.012) and BMI ≥25 kg/m^2^ (HR 1.82 [95% CI 1.137–2.898]; *p* = 0.012). All variables used in the multivariate analysis appear in Table 2.

**TABLE 2:** Multivariate analysis of VTE in the whole study group. Overall death was used as a competing event

	HR	95% CI	*p* value
Age (years) at operation	1.000	0.964–1.012	0.322
Stage III vs. I–II	0.847	0.487–1.48	0.559
Tumor size (mm)	1.011	0.993–1.03	0.223
BMI ≥25 kg/m^2^	1.815	1.137–2.898	**0.012**
Preoperative oncologic therapy	1.897	1.167–3.082	**0.010**
Adjuvant therapy	0.878	0.510–1.512	0.639
Perivascular invasion	1.253	0.752–2.086	0.385

Bold indicates statistical significance values (*p* < 0.05).

Abbreviations: CI, confidence interval; HR, hazard ratio.

We analyzed the VTE‐associating factors of preoperative chemotherapy patients separately (Table 3). Preoperative BMI ≥30 kg/m^2^ associated with an elevated risk of VTE (HR 3.04; 95% CI 1.289–7.162; *p* = 0.011), whereas BMI ≥25 kg/m^2^ did not associate with VTE. In contrast to all patients analyzed together, in the analysis of preoperative chemotherapy patients, the prior thrombotic disease with the use of preoperative anticoagulation increased the risk of VTE (HR 2.83; 95% CI 1.128–7.080; *p* = 0.027). Analyzing those five patients with a prior VTE preoperatively, the risk for a new VTE was great and two of them (40%) had a new VTE postoperatively. Almost every preoperative chemotherapy patient with VTE experienced disease recurrence at some point during the follow‐up period (*n* = 26 [92.9%]) compared with patients with no VTE (*n* = 42 [64.6%]; *p* = 0.005).

**TABLE 3:** The factors associated with VTE in the preoperative oncologic therapy patients

*n* (%)	VTE after surgery		*p* value
	Yes	No	
	28	65	
Gender			
Female	16 (57.1)	31 (47.7)	
Male	12 (42.9)	34 (52.3)	0.403
Age (years) at operation			
≥65	12 (42.9)	33 (50.8)	
<65	16 (57.1)	32 (49.2)	0.484
Respiratory disease			
Yes	4 (14.3)	1 (1.5)	
No	24 (85.7)	64 (98.5)	**0.027**
Cardiovascular disease			
Yes	5 (17.9)	6 (9.2)	
No	23 (82.1)	59 (90.8)	0.297
Medication			
ASA			
Yes	7 (25.0)	12 (18.5)	
No	21 (75.0)	53 (81.5)	0.473
Statin			
Yes	7 (25.0)	12 (18.5)	
No	21 (75.0)	53 (81.5)	0.473
Preoperative anticoagulant			
Yes	6 (21.4)	5 (7.7)	
No	22 (78.6)	60 (92.3)	0.081
Preoperative BMI ≥25 kg/m^2^			
Yes	17 (60.7)	28 (43.1)	
No	11 (32.3)	37 (56.9)	0.118
Preoperative BMI ≥30 kg/m^2^			
Yes	7 (25.0)	4 (6.2)	
No	21 (75.0)	61 (93.8)	**0.010**
Type of surgery			
Pancreaticoduodenectomy	24 (85.7)	60 (92.3)	
Distal pancreatetctomy	4 (14.3)	3 (4.6)	
Total pancreatetctomy	0 (0.0)	2 (3.1)	0.175
Vascular reconstruction			
Yes	16 (57.1)	39 (60.0)	
No	12 (42.9)	26 (40.0)	0.797
Grade			
1	5 (17.9)	9 (13.8)	
2	19 (67.9)	38 (58.4)	
3	3 (10.7)	15 (23.1)	0.555
Missing^a^	4		
Perineural invasion			
Yes	22 (78.6)	43 (66.2)	
No	6 (21.4)	21 (32.3)	0.270
Missing^a^	1		
Perivascular invasion			
Yes	11 (32.3)	19 (29.2)	
No	17 (60.7)	45 (69.2)	0.366
Missing^a^	1		
Stage			
IA	3 (10.7)	6 (9.2)	
IB	7 (25.0)	17 (26.2)	
IIA	3 (10.7)	3 (4.6)	
IIB	8 (28.6)	24 (36.9)	
III	7 (25.0)	14 (21.5)	0.901
Missing^a^	1		
Resection margin			
R0	24 (85.7)	47 (72.3)	
R1	4 (14.3)	18 (27.7)	0.193
Adjuvant therapy			
Yes	21 (75.0)	50 (76.9)	
No	7 (25.0)	14 (21.5)	0.742
Missing^b^	1		
Disease recurrence			
Yes	26 (92.9)	42 (64.6)	
No	2 (7.1)	23 (35.4)	**0.005**

Bold indicates statistical significance values (*p* < 0.05).

^a^ Complete or nearly complete response to preoperative therapy.

^b^ Data could not be found.

### 3.2 Venous thromboembolism and overall survival

**FIGURE 3:**
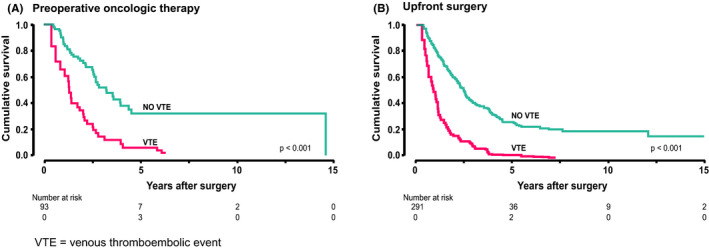
Survival analyses for preoperative oncologic therapy and upfront surgery patients. (A) Survival analysis describing the association between venous thromboembolism after surgery and worse overall survival in preoperative oncologic therapy patients. (B) Survival analysis describing the association between venous thromboembolism after surgery and worse overall survival in upfront surgery patients VTE = venous thromboembolic event

In the overall survival analysis, VTE served as a significant risk factor for both the preoperative chemotherapy (HR 3.25; 95% CI 2.36–4.44; *p* < 0.001) and upfront surgery groups (HR 2.91; 95% CI 1.74–4.85; *p* < 0.001; Figure 3a,b). Additionally, analyzing the whole study group if a patient was operated in 2010 or later, the overall survival was favorable (HR 0.77, 95% CI 0.61–0.97, *p* = 0.029). In the preoperative oncologic therapy group, other factors associated with overall survival in univariate analysis were age at operation (HR 1.03, 95% CI 1.005–1.076, *p* = 0.024) and adjuvant therapy (HR 0.44 95% CI 0.26–0.75 *p* = 0.003). In the upfront surgery group, the associating factors were tumor size (mm) (HR 1.01, 95% CI 1.00–1.02, *p* < 0.001), R0 resection (HR 0.73, 95% CI 0.55–0.98, *p* = 0.035), adjuvant therapy (HR 0.68, 95% CI 0.52–0.91, *p* = 0.008), and perivascular invasion (HR 1.85, 95% CI 1.40–2.44, *p* < 0.001). All the overall univariate analyses are seen in Table S2. We performed a multivariate analysis of overall survival for both groups. In the preoperative oncologic therapy group, risk factors consisted of an older age at surgery (*p* = 0.030), lack of adjuvant therapy (*p* = 0.003), and VTE after surgery (*p* = 0.009). In the upfront surgery group, the risk factors consisted of a higher stage (*p* = 0.001), bigger tumor size (mm) (*p* = 0.026), lack of adjuvant therapy (*p* < 0.001), perivascular invasion (*p* = 0.005), grade 3 versus grade 2 or grade 1 (*p* < 0.001) and VTE after surgery (*p* < 0.001). All factors used in the multivariate analyses appear in Tables S3 and S4, respectively. Furthermore, analyzing the whole study group, if a patient had a splanchnic vein thrombosis, the overall survival was even worse compared with other types of thrombosis (HR 1.75, 95% CI 1.06–2.87, *p* = 0.028). The median time from VTE to death for preoperative chemotherapy and upfront surgery patients was 13.2 (interquartile range [IQR] 3.2–17.6) and 10.2 (IQR 1.7–15.1) months, respectively (*p* = 0.353).

### 3.3 Venous thromboembolism and disease recurrence

During the follow‐up period, disease reoccurred in 298 (77.6%) patients. Among these, 81 (27%) developed VTE. Among the 86 (22%) patients with no disease recurrence during the follow‐up period, 6 (7.0%) developed VTE (*p* < 0.001). Among the 81 patients with disease recurrence and VTE, 58 (72%, 95% CI 60.5–81.1) experienced VTE after disease recurrence. This was significantly more than 50% (*p* < 0.001), the statistically estimated proportion of patients with VTE after disease recurrence with respect to the total follow‐up period, assuming the probability for VTE would remain constant over time. The median time for disease recurrence was 368 days (95% CI 318–418 days).

## 4 DISCUSSION

We found that preoperative oncologic therapy represents an independent risk factor for VTE following pancreatic cancer surgery. The elevated risk in patients receiving preoperative chemotherapy compared with upfront surgery patients was assessed through the first 2 years following surgery. Specifically, in patients receiving preoperative oncologic therapy, the incidence of VTE events climbed fastest during the first 2 years postoperatively, where after incidence plateaued. The majority of VTE events in our study consisted of lower‐extremity deep vein thrombosis, pulmonary embolisms, and splanchnic vein thrombosis, all of which are common thrombosis sites for cancer patients.^6^, ^30^

Surgery and chemotherapy are both well‐established risk factors for VTE.^20^, ^21^, ^24^ For patients receiving chemotherapy followed by surgical resection, chemotherapy and surgery carry a compounded risk for VTE. Thus, the postoperative period is of special interest. To date, only a few studies have analyzed the effect of preoperative chemotherapy on venous thrombosis. Krepline et al. (2016) observed a 10% incidence of VTE during neoadjuvant therapy. However, their analysis did not include the postoperative period.^28^ Boone et al. (2019) showed that for 20% of patients receiving preoperative chemotherapy, VTE occurred at some point following the initiation of chemotherapy through the 90‐day postoperative period. For 70% of these patients (14% of all), VTE occurred during the postoperative period rather than during preoperative chemotherapy, emphasizing the relevance of the postsurgical period when analyzing VTE.^29^

To our knowledge, this is the first study to report an elevated risk of VTE among patients receiving preoperative oncologic therapy compared with patients undergoing upfront surgery. This increased risk can be observed for up to 2 years following surgery. There are multiple studies describing the mechanisms of VTE in PDAC. Tissue factor, a primary initiator of the coagulation cascade, is widely expressed by the PDAC tissue and the best‐characterized factor associated with thrombosis. Tissue factor is also associated with tumor invasion, metastasis, and poor prognosis in pancreatic cancer.^31^, ^32^, ^33^, ^34^ This could partly explain the burden of VTE in patients with the metastasized disease. Also, an increase in the tumor marker CA 19‐9 has been described to predict a VTE incidence in PDAC patients while also being a reliable sign of disease recurrence.^17^, ^35^ Furthermore, increased levels of coagulation factor FVIII, also shown to contribute to the risk of VTE,^36^ have been documented in PDAC.^18^, ^19^

The mechanisms of the long‐term increased risk in preoperatively treated patients, however, remain incompletely understood. Patients receiving preoperative chemotherapy developing VTE might have had a more advanced stage prior to treatment, which may partly increase the risk for VTE. Preoperatively administered chemotherapy may disrupt the tumor tissue, leading to dissemination and long‐term coagulation activity. This, combined with risk factors, such as major abdominal surgery, hospitalization, immobilization, and possible comorbidities, seems to enhance the risk for a longer time than previously understood. In this study, upfront surgery patients also suffered from a marked burden of VTE. These findings lead to the question of whether all patients undergoing pancreatic cancer surgery, especially those receiving preoperative chemotherapy, should receive longer postoperative anticoagulation. At Helsinki University Hospital, a 4‐week postoperative low‐molecular weight thromboprophylaxis for surgically treated pancreatic cancer is strongly established. However, VTE incidence remained frequent throughout the first 2 years following surgery.

Among patients receiving preoperative oncologic therapy, a series of characteristics strongly associated with an even higher risk of VTE. For instance, 11 (12%) patients suffered from atrial fibrillation, heart valve replacement, or prior thrombotic disease and they had prior anticoagulation. These patients had a 2.8‐fold risk of VTE compared with patients without previous indications for anticoagulation. Especially, patients with prior VTE before surgery were at high risk of having a new VTE after surgery. In these patients with preoperative thrombogenic condition, the cumulative risk factors including the preoperative thrombogenic condition, PDAC, abdominal surgery, and hospitalization overlayed and overrode the preventing effect of anticoagulation. Those patients with a prior VTE preoperatively as a paraneoplastic feature were at especially high risk of having a new postoperative thrombosis despite the anticoagulant. This same association between prior thrombotic disease and greater VTE risk was not observed among upfront surgery patients, 23 (8%) of whom received preoperative anticoagulation emphasizing the impact of preoperative chemotherapy on VTE.

In our study, obesity was associated with VTE among all patients and in the preoperative chemotherapy group as well. Unsurprisingly, obesity represents an established risk factor for VTE in cancer patients.^8^ A similar finding focusing on neoadjuvant‐treated pancreatic cancer patients was found in a previous study, where BMI >30 kg/m^2^ represented an independent risk factor for VTE during the 90‐day postoperative period.^29^ In that study, VTE was also associated with vascular resection and adjuvant therapy, an association that did not hold in our study.^29^In addition, a higher stage is a known risk factor for VTE.^27^ But, in our study, a higher stage failed to reach a significant level of association. Preoperative chemotherapy patients had generally lower stage compared with upfront surgery patients, possibly confounding the lack of a difference in the VTE rate between stages. In addition, we excluded stage IV patients. Patients treated before 2010 received a shorter, less than 4‐week anticoagulation postoperatively, but in our study, this was not a significant risk factor for VTE.

Preoperative ASA and statin administration did not associate with VTE in either of the groups. ASA is an established treatment of atherosclerotic diseases such as coronary artery disease.^37^, ^38^ Some studies suggested benefits to ASA in the prevention of VTE^39^, ^40^ but did not focus on cancer patients. Furthermore, the statin is widely used to prevent atherosclerotic events. Approximately 25% (*n* = 97) of all medication regimens for the patients in our study included a statin. A study among 170,459 cancer patients compared the effect of a statin on deep vein thrombosis, finding no difference in outcomes between statin use and no statin,^41^ similar to our own observations.

Furthermore, we found that overall survival was significantly lower among patients with VTE in both, the preoperative chemotherapy and upfront surgery groups. The median time from VTE to death was 10 to 13 months in both groups, rendering VTE an equal burden for all pancreatic cancer surgical patients. VTE is known to reduce overall survival in cancer^1^, ^42^ and also in pancreatic cancer treated with preoperative chemotherapy.^29^ To the best of our knowledge, this is the first study to report a comparison with upfront surgery patients. Additionally, patients with splanchnic vein thrombosis compared with other VTE had an even worse overall survival. In previous studies, splanchnic vein thrombosis in pancreatic cancer has been associated with metastasized disease and unfavorable survival.^30^, ^43^

Analyzing the whole study group, the surgery date after 2010 versus before 2010 was favorable. A previous study demonstrated the improved survival in PDAC patients treated after 2000 versus before 2000,^44^ and based on our study, the improvement of survival in PDAC patients has continued. The use of preoperative oncologic treatments has increased in time which may partly explain the difference. Also, at our hospital, the patients treated in 2010 or after routinely received longer postoperative anticoagulation of 4 weeks. In our study, the longer anticoagulation was not associated with VTE risk, but some studies suggest that longer anticoagulation is independently associated with improved survival.^45^

Other factors associated with overall survival in multivariate analyses were age and adjuvant therapy in the preoperative oncologic therapy group and higher stage, tumor size, perivascular invasion, higher grade, and adjuvant therapy in the upfront surgery group. The patients included in the preoperative oncologic therapy group were more evenly distributed by age, with 52% younger than 65 years, whereas in the upfront surgery group only 39% were younger than 65 years. This may explain the difference in age association between the groups. Preoperative oncologic therapy patients had generally smaller stages, and grade which may explain why stage, tumor size, and tumor grade only associated with overall survival in the upfront surgery group. In the upfront surgery group, R0 resection was associated with overall survival in univariate analysis but not in multivariate analysis. In the preoperative oncologic therapy group, R0 resection did not associate with overall survival. A previous study suggests that margin positivity is not associated with survival in patients receiving preoperative chemotherapy.^46^

We investigated the relationship between disease recurrence and VTE, finding that recurrence occurred in most patients, with 27% developing VTE during the follow‐up period. VTE incidence was significantly more frequent in patients with disease recurrence compared with disease‐free patients. Among patients with both disease recurrence and VTE, 72% experienced VTE after disease recurrence, exceeding the prediction of 50%. Among patients without disease recurrence, VTE remained relatively rare (7%), supporting the previous results of the association between VTE and CA 19‐9 and between tissue factor, VTE, and disease progression.^31^, ^32^, ^33^, ^34^, ^35^ The association between VTE and poor disease‐free survival in pancreatic cancer patients receiving preoperative chemotherapy was previously demonstrated.^29^ However, in our study, VTE occurred following disease progression in 72% of the cases, complicating the survival analysis model. Thus, we applied the one‐sample binomial test comparing the statistically predicted proportion to the observed proportion of patients. These findings showed that the risk of VTE was lower in disease‐free pancreatic cancer patients. Following disease progression, the risk of VTE markedly increases. The median time for disease recurrence was approximately 1 year. The recurrence‐associated VTE partially explains the sustained risk for VTE in both patient groups.

One strength of our study lies in its relatively large, consecutive cohort of surgically treated patients with histologically verified PDAC. Furthermore, the preoperative chemotherapy and upfront surgery patient groups were relatively similar to one another in terms of patient characteristics.

We also acknowledge weaknesses in our study. Due to the retrospective model and relatively large cohort, the treatment modalities varied in time. In 2010, the 4‐week postoperative low‐molecular weight thromboprophylaxis for pancreatic cancer surgery was established at our hospital. Previously (2000–2010), postoperative anticoagulation was administered only during hospitalization. Thus, 58 (20%) upfront surgery and 8 (9%) preoperative chemotherapy patients treated between 2000 and 2010 received a shorter than 4‐week postoperative thromboprophylaxis. This shorter thromboprophylaxis, however, did not emerge as a risk factor for VTE. Due to the retrospective model of our study, we were unable to collect data on some known VTE risk factors including family history and smoking. However, diagnosed hereditary prothrombotic conditions were excluded from the study to avoid confounding effects.

### 4.1 Conclusion

Preoperative oncologic therapy represents an independent risk factor for VTE following pancreatic cancer surgery not only during the immediate postoperative period but up to 2 years after surgery. Obesity, preoperative anticoagulation due to prior VTE or cardiovascular conditions, and disease recurrence increase that risk further. Both upfront surgery and preoperative chemotherapy patients with VTE have significantly and similarly lower overall survival.

## CONFLICT OF INTERESTS

The authors declare no conflicts of interest.

## AUTHORS’ CONTRIBUTIONS

AE wrote the first draft of the manuscript. HS, CH, and RL supervised and took part in the design of the study. All authors took part in the preparation of the manuscript. AE and NM collected the patient data. HM and AE completed the statistical analysis.

## ETHICS APPROVAL

This study complies with the Declaration of Helsinki and was approved by the Surgical Ethics Committee of the Helsinki University Hospital (Dnro HUS 226/E6/06, extension TMK02 §66 17 April 2013).

## Supporting information

Table S1‐S4

## Data Availability

Data supporting the results of this study are available upon request to the corresponding author.
