# Supplementary material for: Preoperative oncologic therapy and the prolonged risk of venous thromboembolism in resectable pancreatic cancer
Source: Cancer Med. 2022 Feb 11;11(7):1605–16. doi: 10.1002/cam4.4397 (PMC8986147; doi:10.1002/cam4.4397)
Supplement: Supplementary file 1 — Table S1‐S4 [file CAM4-11-1605-s001.docx]

**Supplementary Data**

**Preoperative oncological therapy and the prolonged risk of venous thromboembolism in resectable pancreatic cancer**

A Eurola^1^, H Mustonen^1^, N Mattila^1^, R Lassila^2,3^, C Haglund^1^, H Seppänen^1^

^1^Department of Surgery, Translational Cancer Medicine Research Program, Faculty of Medicine, University of Helsinki and Helsinki University Hospital, Helsinki, Finland

^2^Department of Coagulation Disorders, Faculty of Medicine, University of Helsinki and Helsinki University Hospital, Helsinki, Finland

^3^HUSLAB Laboratory Services, Clinical Chemistry, Helsinki, Finland

| **Supplementary Table 1.** The time-dependent variables used in the univariate analyses of venous thromboembolism following surgery in the entire sample. The variables presented here yielded no statistically significant results. | | | | |  |
| --- | --- | --- | --- | --- | --- |
|  |  |  |  |  |  |
|  |  |  |  |  |  |
|  | **HR** | **95% CI** | | **p - value** |  |
| **Gender** | 0.874 | 0.572 | 1.333 | 0.531 |  |
| **Age at surgery ≥65 years** | 0.948 | 0.590 | 1.521 | 0.070 |  |
| **Respiratory disease** | 1.220 | 0.531 | 2.806 | 0.639 |  |
| **Cardiovascular disease** | 0.801 | 0.446 | 1.378 | 0.423 |  |
| **Medication** |  |  |  |  |  |
| Preoperative ASA | 1.008 | 0.606 | 1.678 | 0.974 |  |
| Preoperative statin | 1.059 | 0.652 | 1.717 | 0.818 |  |
| Preoperative anticoagulant | 0.863 | 0.398 | 1.870 | 0.709 |  |
| Postoperative anticoagulant < four weeks | 0.815 | 0.329 | 2.019 | 0.658 |  |
| **Vascular reconstruction** | 1.381 | 0.901 | 2.115 | 0.138 |  |
| **Type of surgery** |  |  |  |  |  |
| Pancreaticoduodenectomy | 1 |  |  |  |  |
| Distal pancreatectomy | 1.628 | 0.916 | 2.893 | 0.097 |  |
| Total pancreatectomy | 2.470 | 0.773 | 7.894 | 0.127 |  |
| **Grade** |  |  |  |  |  |
| 1 | 1 |  |  |  |  |
| 2 | 1.341 | 0.746 | 2.410 | 0.327 |  |
| 3 | 1.339 | 0.627 | 2.855 | 0.450 |  |
| **Perineural invasion** | 1.433 | 0.813 | 2.524 | 0.213 |  |
| **Perivascular invasion** | 1.317 | 0.850 | 2.040 | 0.217 |  |
| **Stage** |  |  |  |  |  |
| IA | 1 |  |  |  |  |
| IB | 1.172 | 0.422 | 3.256 | 0.760 |  |
| IIA | 2.891 | 0.834 | 10.019 | 0.094 |  |
| IIB | 1.777 | 0.700 | 4.513 | 0.226 |  |
| III | 1.934 | 0.733 | 5.099 | 0.182 |  |
| **Resection margin** | 0.999 | 0.610 | 1.635 | 0.996 |  |
| **Tumor size (mm)** | 1.009 | 0.994 | 1.024 | 0.208 |  |
| **Adjuvant therapy** | 0.987 | 0.608 | 1.609 | 0.958 |  |
| Abbreviations: HR, hazard ratio; CI, confidence interval. | | | |  |  |

| **Supplementary Table 2.** The variables used in the univariate analysis of overall survival in the preoperative oncological therapy and upfront surgery group. | | | | | | | | |  |
| --- | --- | --- | --- | --- | --- | --- | --- | --- | --- |
|  |  |  |  |  |  |  |  |  |  |
|  | Preoperative oncological therapy | | | | Upfront surgery | | | |  |
|  | HR | 95% CI | | p - value | HR | 95% CI | | p - value |  |
| **Gender** | 0.899 | 0.702 | 1.151 | 0.399 | 0.920 | 0.808 | 1.048 | 0.208 |  |
| **Age (years) at surgery** | 1.035 | 1.004 | 1.067 | **0.024** | 1.001 | 0.985 | 1.017 | 0.881 |  |
| **Respiratory disease** | 1.507 | 0.540 | 4.204 | 0.433 | 1.277 | 0.796 | 2.048 | 0.311 |  |
| **Cardiovascular disease** | 1.102 | 0.542 | 2.236 | 0.789 | 0.863 | 0.637 | 1.170 | 0.343 |  |
| **Medication** |  |  |  |  |  |  |  |  |  |
| Preoperative ASA | 0.829 | 0.442 | 1.556 | 0.560 | 0.923 | 0.674 | 1.264 | 0.618 |  |
| Preoperative statin | 0.724 | 0.386 | 1.359 | 0.315 | 1.120 | 0.835 | 1.503 | 0.451 |  |
| Preoperative anticoagulant | 1.630 | 0.773 | 3.436 | 0.199 | 0.683 | 0.410 | 1.137 | 0.142 |  |
| **Vascular reconstruction** | 1.119 | 0.676 | 1.852 | 0.661 | 1.330 | 0.100 | 1.770 | 0.050 |  |
| **Grade** |  |  |  |  |  |  |  |  |  |
| 1 | 1 |  |  |  | 1 |  |  |  |  |
| 2 | 0.982 | 0.501 | 1.923 | 0.958 | 0.982 | 0.501 | 1.923 | 0.958 |  |
| 3 | 1.010 | 0.455 | 2.239 | 0.980 | 1.010 | 0.455 | 2.239 | 0.980 |  |
| **Perineural invasion** | 1.705 | 0.952 | 3.051 | 0.072 | 1.411 | 0.993 | 2.005 | 0.054 |  |
| **Perivascular invasion** | 1.456 | 0.854 | 2.481 | 0.167 | 1.845 | 1.396 | 2.440 | **< 0.001** |  |
| **Stage** |  |  |  |  |  |  |  |  |  |
| IA | 1 |  |  |  | 1 |  |  |  |  |
| IB | 0.722 | 0.302 | 1.725 | 0.465 | 0.722 | 0.302 | 1.725 | 0.465 |  |
| IIA | 0.846 | 0.253 | 2.826 | 0.787 | 0.846 | 0.253 | 2.826 | 0.787 |  |
| IIB | 1.012 | 0.450 | 2.278 | 0.976 | 1.012 | 0.450 | 2.278 | 0.976 |  |
| III | 1.077 | 0.454 | 2.553 | 0.866 | 1.077 | 0.454 | 2.553 | 0.866 |  |
| **Resection margin** | 1.490 | 0.831 | 2.672 | 0.181 | 1.362 | 1.021 | 1.819 | **0.035** |  |
| **Tumor size (mm)** | 1.017 | 0.996 | 1.039 | 0.109 | 1.017 | 1.007 | 1.027 | **< 0.001** |  |
| **Adjuvant therapy** | 0.440 | 0.258 | 0.749 | **0.002** | 0.684 | 0.516 | 0.906 | **0.008** |  |
| Abbreviations: HR, hazard ratio; CI, confidence interval. | | | | |  |  |  |  |  |

| **Supplementary Table 3.** Variables used in the multivariate analysis of overall survival among preoperative oncological therapy patients. Venous thromboembolism is a time-dependent variable. | | | | |  |
| --- | --- | --- | --- | --- | --- |
|  |  |  |  |  |  |
|  | HR | 95% CI | | p - value |  |
| Age (years) at surgery | 1.045 | 1.011 | 1.080 | **0.030** |  |
| Stage III vs. I–II | 0.789 | 0.406 | 1.533 | 0.871 |  |
| Tumor size (mm) | 1.012 | 0.991 | 1.034 | 0.246 |  |
| Adjuvant therapy | 0.306 | 0.164 | 0.571 | **0.003** |  |
| R0 resection | 2.020 | 1.051 | 3.881 | 0.073 |  |
| Perivascular invasion | 1.294 | 0.699 | 2.393 | 0.634 |  |
| Grade 3 vs. 1-2 | 1.169 | 0.614 | 2.224 | 0.914 |  |
| Venous thromboembolism | 3.747 | 2.095 | 6.700 | **0.009** |  |
| Abbreviations: HR, hazard ratio; CI, confidence interval | | |  |  |  |
|  |  |  |  |  |  |
|  |  |  |  |  |  |
|  |  |  |  |  |  |
| **Supplementary Table 4.** Variables used in the multivariate analysis of overall survival among upfront surgery patients. Venous thromboembolism is a time-dependent variable. | | | | |  |
|  |  |  |  |  |  |
|  | HR | 95% CI | | p - value |  |
| Age (years) at surgery | 1.002 | 0.984 | 1.012 | 0.649 |  |
| Stage III vs. I–II | 1.695 | 1.202 | 2.391 | **0.001** |  |
| Tumor size (mm) | 1.013 | 1.002 | 1.025 | **0.026** |  |
| Adjuvant therapy | 0.596 | 0.430 | 0.827 | **<0.001** |  |
| R0 resection | 0.974 | 0.696 | 1.361 | 0.853 |  |
| Perivascular invasion | 1.488 | 1.091 | 2.030 | **0.005** |  |
| Grade 3 vs. 1-2 | 1.578 | 1.145 | 2.170 | **<0.001** |  |
| Venous thromboembolism | 2.947 | 2.087 | 4.159 | **< 0.001** |  |
| Abbreviations: HR, hazard ratio; CI, confidence interval | | |  |  |  |
